# Machine learning algorithms for the evaluation of risk by tick-borne pathogens in Europe

**DOI:** 10.1080/07853890.2024.2405074

**Published:** 2024-09-30

**Authors:** Agustín Estrada-Peña, José de la Fuente

**Affiliations:** a Department of Animal Health, Faculty of Veterinary Medicine, University of Zaragoza, Zaragoza, Spain; b SaBio (Health and Biotechnology), Instituto de Investigación en Recursos Cinegéticos IREC-CSIC-UCLM-JCCM, Ciudad Real, Spain; c Department of Veterinary Pathobiology, Center for Veterinary Health Sciences, Oklahoma State University, Stillwater, OK, USA

**Keywords:** Tick habitat suitability, modelling, Europe, hosts distribution, landscape features, machine learning algorithms, human health

## Abstract

**Background:**

Tick-borne pathogens pose a major threat to human health worldwide. Understanding the epidemiology of tick-borne diseases to reduce their impact on human health requires models covering large geographic areas and considering both the abiotic traits that affect tick presence, as well as the vertebrates used as hosts, vegetation, and land use. Herein, we integrated the public information available for Europe regarding the variables that may affect habitat suitability for ticks and hosts and tested five machine learning algorithms (MLA) for predicting the distribution of four prominent tick species across Europe.

**Materials and methods:**

A grid of cells 20 km in diameter was prepared to cover the entire territory, containing data on vegetation, points of water, habitat fragmentation, forest density, grass extension, or imperviousness, with information on temperature and water deficit. The distribution of the hosts (162 species) was modelled and included in the dataset. We used five MLA, namely, Random Forest, Neural Networks, Naive Bayes, Gradient Boosting, and AdaBoost, trained with reliable coordinates for *Ixodes ricinus*, *Dermacentor reticulatus*, *Dermacentor marginatus*, and *Hyalomma marginatum* in Europe.

**Results:**

Both Random Forest and Gradient Boosting best predicted ticks and host environmental niches. Our results demonstrate that MLA can identify trait-matching combinations of environmental niches. The inclusion of land cover and land use variables has a superior capacity for predicting areas suitable for ticks, compared to classic methods based on the use of climate data alone.

**Conclusions:**

Flexible MLA-driven models may offer several advantages over traditional models. We anticipate that these results may be extrapolated to other regions and combinations of tick-vertebrates. These results highlight the potential of MLA for inference in ecology and provide a background for the evolution of a completely automatized tool to calculate the seasonality of ticks for early warning systems aimed at preventing tick-borne diseases.

## Introduction

Ticks and tick-borne pathogens (TBPs) constitute major threats to human health worldwide [1]. Ecologists have long suspected that species are more likely to interact if their traits match in a particular manner [2, 3]. It is known that ticks and vertebrates interact in different ways to improve the circulation of TBPs, which is commonly disregarded when modelling the prevalence of these diseases [4, 5]. Advances have been made to understand the role of some hosts in the circulation of TBPs [6] in field surveys of variable size because of the difficulties resulting from large-scale surveys [7, 8]. On the other hand, modelling has been used to map the potential distribution of TBPs without considering that their patterns of circulation depend on the availability of competent reservoirs [9, 10]. The importance of landscape features in the habitat suitability of ticks is well supported by field studies [11–15]. While commonly neglected, the relative composition of the communities of vertebrates may have a pivotal role in modelling the distribution of ticks and/or TBPs [16, 17]. Climate, landscape, and biotic (hosts-derived) explanatory variables have only been partially integrated into datasets involving large territories, such as continents [18]. Despite the exponential growth in data availability, broader interoperability among datasets is still necessary. This would be applicable not only to the basic knowledge of tick ecology but also for understanding the probable distribution of ticks transmitting pathogens from solid, reliable, open-source, and curated sources of data. Such a framework should have an adequate resolution to capture the regional events impacting the distribution of both ticks and TBPs, and we propose a grid of homogeneously sized polygons accommodating the regional traits acting on ticks and vertebrate demographic processes.

Modelling methods used for ticks are commonly restricted to a subset of the methods currently available [19–21]. The big data revolution has led to ecological studies of arthropod-borne pathogens, together with a panoply of modelling methods collectively known as Machine Learning Algorithms (MLA). Climate data have been commonly used for tick modelling approaches, sometimes with the inclusion of an index of vegetation stress (the NDVI or normalized difference vegetation index). Other explanatory variables, such as those derived from the landscape composition and topology, or the presence/absence of vertebrates used as competent reservoirs or propagation hosts, are not a common part of such a modelling framework.

In this study, we addressed the challenge of assembling and providing an open-access dataset that includes the most important variables that define ticks and hosts environmental niches. We then compared the ability of several MLA to select the necessary variables to predict the niche of both ticks and vertebrates in Europe. In addition to releasing the first European dataset of tick and host distributions, together with climate, landscape, and biotic variables, we aimed to explore the outcomes of methods that have not been tested before in this context and to check if the distribution of the vertebrates that ticks feed improves the modelling of the tick’s distribution. These results advance the prediction of the threats associated with tick-borne diseases in Europe and have possible applications in other regions.

## Material and methods

### Background: structure of the dataset

The Copernicus Land Monitoring System hosts the greatest and most detailed datasets of land use and land cover features available for Europe (https://www.esa.int/Applications/Observing_the_Earth/Copernicus/Europe_s_Copernicus_programme). It contains data of interest to identify the variables that shape the niche of ticks and vertebrates. Hence, we approached the integration of data for the joint modelling of the distribution of vertebrates and ticks in Europe using (a) a large set of climate data, (b) a set of data regarding the landscape, such as several features of vegetation, its fragmentation, dominance, land use, and distance to critical features known to affect tick presence, and (c) the distribution of vertebrates in the European territory that is also modelled from available records and the variables mentioned below. We explored the power of several MLA selecting the best combinations of such a large set of variables to produce the best description of the tick/vertebrate habitats (Table 1). The aim of MLA is to assess the distribution and habitat suitability of both ticks and hosts. We used the term ‘habitat’ to name the combination of abiotic (climate), biotic (vertebrate hosts), and landscape features, such as amount of forest and fragmentation among others that integrate the target territory. Habitat suitability is thus a continuous numeric outcome, that shapes the territory in which a species (tick or vertebrate) may be present or absent. Existing environmental data for Europe (such as forest composition, grass communities, or points of water) are commonly delivered in a raster format, at high resolution (even at 10 m/pixel for the whole continent) on the official European Environmental Agency website (https://www.eea.europa.eu/data-and-maps) or on the Ecodatacube website (https://stac.ecodatacube.eu/). The dataset presented here comes in part from the official links mentioned above; it is complemented by climate interpolated data.

**Table 1.** The abiotic variables (climate, vegetation, and landscape features) included in the dataset for modelling the environmental niche of four species of ticks and their vertebrate hosts in Europe.

Name of the variable	Meaning	Obtained from	Available at	Comments
EEA median	Median value of the vegetation category (the most represented value in the target cell)	Ecosystems Types of Europe V3.1 eea_r_3035_100_m_etm-terrestrial-c_2012_v3-1_r00.tif	https://sdi.eea.europa.eu/catalogue/srv/api/records/7c0cf3f2-ab54-4cd0-a635-b322df7197f6	A document ‘Ecosystem_mapping_v3_1.pdf’ explains all the details. Available in the link provided. The dataset includes 52 categories of vegetation.
EEA minority	The category of vegetation of the smallest patch in the target cell	Ecosystems Types of Europe V3.1 eea_r_3035_100_m_etm-terrestrial-c_2012_v3-1_r00.tif		
EEA majority	The category of vegetation of the largest patch in the target cell	Ecosystems Types of Europe V3.1 eea_r_3035_100_m_etm-terrestrial-c_2012_v3-1_r00.tif		
EEA variety	The number of different categories of vegetation in the target cell of the grid	Ecosystems Types of Europe V3.1 eea_r_3035_100_m_etm-terrestrial-c_2012_v3-1_r00.tif		
FTY	Type of forest (coniferous, broadleaf or mixed)	FTY_2018_100m_eu_03035_V1_0.tif	https://land.copernicus.eu/pan-european/high-resolution-layers/forests/forest-type-1	The indicator is a measure of the dominant type of forest in each tile
FTY_buffer	Average distance of all the pixels in a cell to the nearest forest patch	FTY_2018_100m_eu_03035_V1_0.tif		The value indicates ‘how near’ is a pixel, in average, from a forest patch of any type
TCDCount	Area of forest	Tree Cover Density TCD_2018_100m_eu_03035_V2_0.tif	https://land.copernicus.eu/pan-european/high-resolution-layers/forests	All the variable beginning with ‘TCD’ refer to ‘tree cover density’, in a value ranging from 0 to 100
TCDSum	Sum of the tree coverage in the area(s) of forest			
TCDvariance	Variance of the tree coverage in the area(s) of forest			
TCDMedian	Median of the tree coverage in the area(s) of forest			
GRSCount	Number of pixels of grass per polygon	GRA_2018_100m_eu_03035_V1_0.tif	https://land.copernicus.eu/en/products/high-resolution-layer-grassland	High Resolution Layer Grassland 2018. Value refers to the area occupied by grass in each cell.
GRSbuffer	Average distance of all the pixels in a cell to the nearest patch of grass.			The value indicates ‘how near’ is a pixel, in average, from a patch of grass of any type
TMAX1	First coefficient of the harmonic regression of the maximum temperature (1990–2020)	TerraClimate Data	https://developers.google.com/earth-engine/datasets/catalog/IDAHO_EPSCOR_TERRACLIMATE	This value also describes the mean multi-annual value of daily maximum temperature
TMAX2	Second coefficient of the harmonic regression of the maximum temperature (1990–2020)			Both values describe the slope of spring and autumn (and the day of beginning according to changes in the time series) as well as the duration of summer and autumn in climatic terms (not calendar dates)
TMAX3	Third coefficient of the harmonic regression of the maximum temperature (1990–2020)			
TMIN1	First coefficient of the harmonic regression of the minimum temperature (1990–2020)	TerraClimate Data	https://developers.google.com/earth-engine/datasets/catalog/IDAHO_EPSCOR_TERRACLIMATE	This variable describes the mean multi-annual value of daily minimum temperature
TMIN2	Second coefficient of the harmonic regression of the minimum temperature (1990–2020)			Both TMIN1 and TMIN2 describe the slope of spring and thus the beginning of spring.
TMIN3	Third coefficient of the harmonic regression of the minimum temperature (1990–2020)			Both TMIN1 and TMIN3 describe the slope of change summer-autumn and thus the beginning of autumn
VPD1	First coefficient of the harmonic regression of the water vapour deficit (1990–2020)	TerraClimate Data	https://developers.google.com/earth-engine/datasets/catalog/IDAHO_EPSCOR_TERRACLIMATE	They describe the mean multi-annual value of vapour pressure deficit
VPD2	Second coefficient of the harmonic regression of the water vapour deficit (1990–2020)			
VPD3	Third coefficient of the harmonic regression of the water vapour deficit (1990–2020)			
Impervious	Impervious surface in the target cell		https://www.eea.europa.eu/data-and-maps/dashboards/imperviousness-in-europe	It provides accounts of land surface sealing status and change in Europe (EEA39 and EU28) for every 3 years between 2006 and 2015
Impervious_buffer	The average distance between impervious surface and any other vegetated patch in the target cell			
Vertebrates (various specific names)	The expected suitability of the cell in the grid for 162 species of vertebrates, reported as hosts for ticks or competent reservoirs for tick-borne pathogen			

Data about the meaning of each abbreviation are included, as well as the link to the original image in raster format, available in the data pool of European Union. Fragmentation of the habitat was not included because existing datasets lack Switzerland and all the Balkans countries, thus affecting the integrity of the dataset. It is known that habitat fragmentation affects the populations of both ticks and vertebrates, but its inclusion in the current dataset has resulted unfeasible.

The basis of the dataset is a set of hexagonal cells that cover the European territory, consisting of 19,291 hexagonal cells with a diameter of 20 km each, covering the target territory; some peripheral cells are smaller because they cover parts of the sea and are therefore cut to follow the coastline. Each cell of the grid is filled with the values of the variables described below, which may have an importance for the delineation of the tick habitat. All the data explained below were entered for each tile using simple overlap GIS methods, allowing the selection of different combinations of variables to build the most reliable models. It is not possible to work with points of raster images because of the large extension of the target territory and because many variables (e.g. landscape features) make sense only in the context of a surface instead of a point. Special care was taken to avoid missing fields while translating the rasters to the polygons dataset; all the 19,291 cells have the complete set of data.

### Climate data

Climate data represent part of the abiotic features that are known to impact the life cycle of ticks. It is widely accepted that adequate combinations of temperature and humidity can delineate the presence/absence/abundance of ticks, provided there are no geographical barriers and suitable hosts are available. In our application, climate data were obtained from the interpolated set known as ‘TerraClimate’ (available at https://www.climatologylab.org/terraclimate.html) at monthly intervals for the period 1990–2022. An interpolated climatology used for tick habitat prediction must include at least data on minimum and maximum temperature and water vapor deficit, or a measure of water in the air. It is known that rainfall is poorly correlated with measures of water in the air [22] and that rainfall may have a local and temporal effect on tick populations. An interpolated climatology dataset avoids gaps produced by ice, clouds, or snow for large periods in satellite imagery (i.e. winter in the northern portions of the target area). The complete TerraClimate time series [23] was summarized as the monthly average of each variable, which was later subjected to harmonic regression. The use of harmonic regression coefficients has been previously validated because they are free of the frequent issues of spatial correlation and multicollinearity between the layers of explanatory variables. The harmonic regression curves have several coefficients. The coefficients of the harmonic regressions retain the annual average values of a variable, the moment and slope of change in spring, the length of summer, and the moment and rate of change in autumn. We used the first three coefficients of the harmonic regression for each climate variable as explanatory layers for predictive mapping with total of nine variables accounting for the maximum and minimum temperature and water vapor deficit. These variables were used scaled at the resolution of the grid. Raster images were overlapped with the grid, and the median value of the raster pixels enclosed by each cell of the grid was obtained and used for modelling (see Table 1).

### Landscape/vegetation and vertebrate host data

Landscape and vegetation data are another major determinant of the distribution of ticks and vertebrates but have rarely been explored (but see [24]). We included a variety of traits, such as the type of forest or grass, dominant land use and/or land cover in each grid cell, the amount, persistence, and extension of aquifers, the fragmentation of the habitat, or the mean distance of each single pixel in the cell to forest, grass, or bare ground. Most of these variables have been associated with tick density in various field studies. The effects of habitat fragmentation (or habitat connectivity) have been demonstrated in ticks, but their impact on the circulation of TBPs has not been explored in a wide area [25]. Table 1 includes the names of all the variables in the final gridded dataset, the link from which the original open-access data were obtained, and its meaning in the context of this study. All these data in their original raster format (some of them well over 10 GB in size) were transferred to each cell of the hexagonal grid using basic GIS overlap techniques.

After building the previous dataset, we addressed the relationships between ticks and their hosts. A high-resolution set of maps displaying the distribution of vertebrates does not exist in Europe, similar to the GAP analysis project for the USA available at https://www.usgs.gov/programs/gap-analysis-project. Therefore, layers with vertebrate distribution in the target territory were prepared *de novo* from the already available information about their presence (see below). Therefore, we obtained information about the distribution of vertebrate species that have been recorded as hosts for the tick(s) and/or reservoirs of pathogen(s). The purpose is 2-fold: building the most complete dataset of traits that could influence the presence/absence/abundance of ticks and interpreting the impact of the inclusion of the suitability of the habitat for vertebrates on tick modelling. We used ∼3 million records of vertebrates that were heterogeneously distributed in the target territory. We developed models to predict the distribution and habitat suitability of these vertebrate species using the same algorithms as for ticks (see below).

### Pre-processing and scaling of data

As mentioned, before building the modelling algorithms and the workflow, we explicitly tested the dataset for missing data. Both dataset and raster data (explanatory variables) were projected with the official European projection (LAEA) and were clipped with the same sea mask. Therefore, the complete set of 19,291 cells had the complete set of explanatory variables without missing data. All the variables were subjected to a rescaling and centering around mean to avoid issues derived from the different scales of the actual data. All the climatic variables were rescaled and centered as it is the most straightforward data transformation and centers and scales a variable to mean zero and standard deviation one. Therefore, it ensures that the criterion for finding linear combinations of the predictors is based on how much variation they explain and therefore improves the numerical stability. Landscape continuous variables (e.g. amount of canopy) were also centered and rescaled. Categorical variables (e.g. type of forest) were not rescaled as before but were turned into continuous by the algorithms before modelling. However, to note, all the data included in the gridded dataset remained with their original values in the released version available as Supplementary Material. We did not want to modify the original values since this could refrain the use of the dataset in other ways. Variables derived from the habitat suitability prediction of vertebrates (continuous values between 0 and 100%) were also centered and rescaled to fit its scale to the previous explanatory variables.

### Evaluation of the habitat suitability for vertebrates in the target territory

The predicted habitat suitability (range: 0–100) of a total of 162 vertebrate species was included in the available dataset. All these species have been reported in the last 40 years as hosts of at least one of the tick species of concern and/or are common reservoirs of pathogens, such as *Borrelia* spp., *Anaplasma phagocytophilum*, or *Rickettsia* spp. The point distribution (coordinates) of the hosts necessary to train the models was the set previously produced for other purposes [18], and it includes about three millions of records, after the removal of records with issues like wrong coordinates (e.g. over the sea). We filtered the species distribution records to ‘couple’ with the resolution of the grid dataset. This is because the reported distribution of vertebrates in the target territory may have a resolution of even a few hundred meters, while the grid has a resolution of 20 km. The point distribution of each vertebrate was overlapped over the grid using a simple spatial query, and we selected the cells of the grid with records of the vertebrate. These cells were marked as positive for the species. Since these surveys are rarely random, we did not consider the number of records of a vertebrate in a cell, and it was turned positive with only one record.

Before defining the workflow of modelling, sets of positive and negative records of each species of vertebrate that are necessary to train the algorithms were prepared. We selected presence cells that were those where the species has been reported. To select absence cells, we adhered to a strategy consisting of the selection of cells in which other vertebrates were reported, confirming the existence of surveys in that cell but the focus species remains unreported. The dataset of absences for each species was built separately in a looping selection of cells. Although these are not real absence sets, preliminary tests confirmed that they perform better than a set of randomly selected pseudo-absences. The pure random selection of pseudo-absences may include climate or landscape conditions that are too extreme for the existence of any vertebrate in our set of species, therefore biasing the choice of the range of explanatory variables, building unreliable models.

Selection of algorithms was based on its ability for modelling other living organisms and its capacity to handle common problems like features election and/or overfitting [11, 13, 15, 18]. We used five MLA: Random Forest, Neural Networks, Naive Bayes, Gradient Boosting, and AdaBoost, that we briefly describe here. Random Forest builds a set of decision trees. Each tree is developed using a bootstrap sample of the training data. When developing individual trees, an arbitrary subset of attributes is drawn (hence the term ‘Random’) from which the best attribute for the split is selected. Random forests use bootstrap sampling and feature bagging to reduce the variance and overfitting of individual trees. Bootstrap sampling means that each tree of decisions is trained on a random subset of data, and feature bagging means that each split is based on a random subset of the features. Random forests can handle both numerical and categorical features and can perform feature importance and handle missing data. The final model is based on a majority vote of individually developed trees in the forest. For Random Forests, we stated the number of trees at 10, with replicate training, without balancing the class distribution. The AdaBoost (‘adaptive boosting’) is a MLA that can be used with other learning algorithms to boost their performance. This is done by tweaking the weak learners. For AdaBoost, we used the option ‘tree’ as base estimator, with 50 estimators and a learning rate of 1, using the SAMME.R as classification algorithm and the linear regression loss function. Gradient Boosting is an MLA for regression and classification problems that produces a prediction model in the form of an ensemble of weak prediction models, typically decision trees. For Gradient Boosting, we used 100 trees, and a learning rate of 0.3, with Lambda = 3. The Neural Networks used 100 neurons in one hidden layer, activated by the rectified linear unit (ReLu) function and the L-BFGS-B solver, with alpha = 0.0001 and 200 maximum iterations. For Naive Bayes, we assumed equiprobable classes (i.e. priors = 1/(number of classes)). These MLA are available in the ‘Orange’ programming environment version 3 (https://orangedatamining.com). Orange is a set of Python routines distributed as an open-access software.

The algorithms calculate the probability of the ­presence/absence of each organism (vertebrate or tick) by selecting the best combination of variables with which such a probability is calculated. The algorithms compare the ‘true’ presence/absence of the modeled organism, building a confusion table (sensitivity and specificity) where the output is a value of the performance of each algorithm for each species over the entire territory. To estimate the skill of the models, we used cross-validation. Cross-validation is a technique for evaluating a model and testing its performance. It compares and selects an appropriate model for the specific predictive modeling problem. The records of each species were divided into a 70% (training set) and a 30% (evaluation set). Each model was trained and then validated against the evaluation set. This was repeated 10 times. Every detail of cross-validation for each algorithm and species can be studied in the scripts provided in the Supplementary Material.

The set of features (explanatory variables) that better explained the known distribution of each species was included in the final model. A maximum of 15 variables were allowed to be selected in the best model for each species, that were selected following the indexes of the model performance including/excluding sets of variables (information provided by the variables). As an indicator of the performance of each MLA, we primarily used the AUC, which is the area under the receiver operating characteristic (ROC) curve. To complete the analysis of performance, we also used the classification accuracy (CA, the proportion of correctly classified records); Precision, which is the proportion of true positives among instances classified as positive; Recall, as the proportion of true positives among all positive instances in the data; F1, which is a weighted harmonic mean of precision and recall; Specificity, which is the proportion of true negatives among all negative instances; and the Matthews correlation coefficient (MCC), which takes into account true and false positives and negatives and is generally regarded as a balanced measure. For model interpretation, the Gain Ratio (a ratio of the information gain and the intrinsic information of the attribute, which reduces the bias towards multivalued features that occur in information gain, the Gini index or the inequality among values of a frequency distribution, and the Chi^2^ or dependence between the feature and the class as measured by the chi-square statistic. All these indexes are accessible in the scripts accompanying this study as Supplementary Material.

In machine learning, overfitting occurs when an algorithm fits too closely or even exactly to its training data, resulting in a model that can’t make accurate predictions or conclusions from any data other than the training data. A model is overfitting the training data when the model performs well on the training data but does not perform well on the evaluation data. We thus watched the evaluation metrics; in case of overfitting, we reduced the feature selection (i.e. the number of explanatory variables selected simultaneously) and/or increased the number of passes on training data. We detected overfitting in four species of vertebrates: two species of the genus *Lacerta* and two species of the family Gliridae. They all have either a major distribution area in southern Palearctic (northern Africa) or eastern Palearctic. The target territory captured only the periphery of their ranges and therefore its niche was inadequately described. These species were removed from the final dataset and the modelling.

We chose the results of the modelling of 11 species of vertebrates as proof-of-concept to show the reliability of the modelling algorithms regarding the reported/modeled distribution of vertebrates. These species are large ungulates (*Alces alces*, *Capreolus capreolus*, *Cervus elaphus*), small-to medium-sized mammals (*Erinaceus europaeus*, *Apodemus sylvaticus*, *Sorex araneus*), small birds (*Parus major*, three species of *Turdus*), and one species of reptiles (*Lacerta lepida*). Three *Turdus* species were selected to demonstrate the power of the proposed methods to map their different areas of distribution. The lacertid is restricted to the Mediterranean region and has been included to demonstrate the ability of the MLA to discern its known niche.

### Evaluation of the habitat suitability for ticks in the target territory

To show the performance of the MLA for ticks, we performed an explicit modelling of the expected occurrence of four species of ticks occurring in Europe, which are of interest to human or animal health, and for which enough geo-referenced field data exist [18], allowing adequate training of models. The dataset includes a total of more than 16,000 reliable records, with an accuracy of geolocation of about 1 kilometer. The four tick species were *Ixodes ricinus*, *Dermacentor marginatus*, *Dermacentor reticulatus,* and *Hyalomma marginatum*. We adhered to the same algorithms, protocols, pre-processing methods, and algorithm tunning described before for the vertebrates (see section 3). Algorithms were allowed to select features derived from the distribution of the vertebrates with explicitly comparing the performance of these variables in relation to the ones commonly used for tick modelling based on climate and landscape features (e.g. [5, 18, 20]). The script for modelling the distribution of these four species of ticks is available as Supplementary Material.

## Results

### Tick species distribution is better modelled using climate and vegetation features

Three types of algorithms performed better than the other two in modelling the environmental niche of the four species of ticks that were tested. These include gradient boosting, random forest, and neural networks. Both AdaBoost and Naive Bayesian performed ∼10% worse, assessed with the indicators of performance. The AUC values for each algorithm and species are included in Table 2. Supplementary Material 1 includes the complete set of indexes of performance of the five algorithms, including CA, F1, Precision, Recall, and MCC (see Methods for definition and interpretation of these indexes). In any case, the AUC was in line with the values of performance detected by other indexes; as mentioned, it was used as the index of reference in the validation of models. The performance of the top MLA for each species for calculating the probability of the presence of each tick species (which was consistently Gradient Boosting) is included in Table 3.

**Table 2.** The AUC values of the best model for each of the four species of ticks tested.

Model AUC	*I. ricinus*	*D. marginatus*	*D. reticulatus*	*H. marginatum*
Gradient boosting	0.980	0.957	0.983	0.982
Random forest	0.966	0.937	0.964	0.968
AdaBoost	0.883	0.785	0.868	0.855
Neural network	0.952	0.923	0.960	0.962
Naive Bayes	0.870	0.847	0.888	0.914

The complete list of performance values for each algorithm is included as Supplementary Material 1.

**Table 3.** The performance of gradient boosting for detecting presence or absence of the four target species of ticks.

Species	Presence (%)	Absence (%)
*I. ricinus*	93.1	85.4
*D. marginatus*	80.9	78.9
*D. reticulatus*	98.2	74.3
*H. marginatum*	98.6	79.1

**Figure 1.**
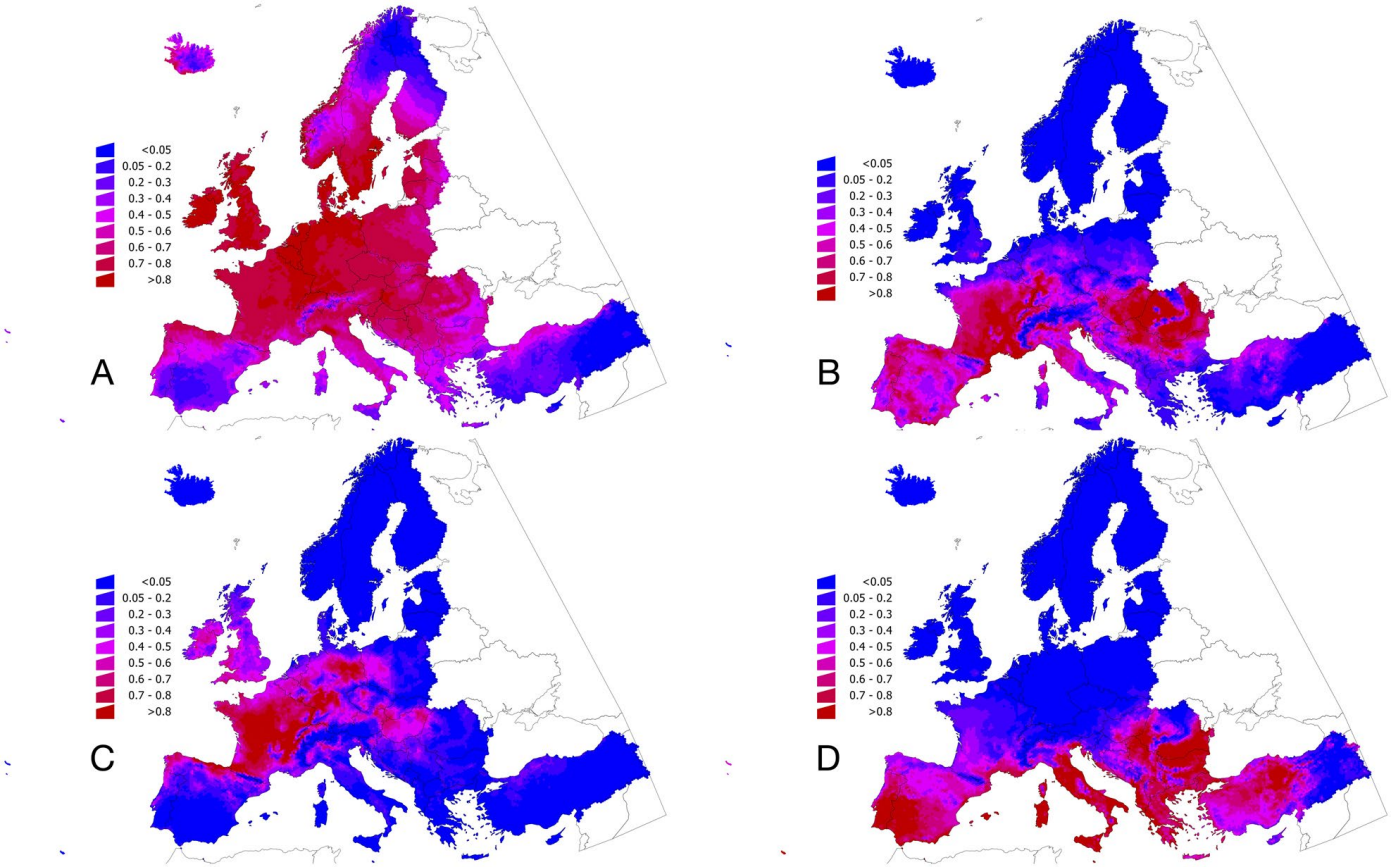
The modelled habitat suitability for the four species of ticks addressed in this study. (A) *Ixodes ricinus*. (B) *Dermacentor reticulatus*. (C) *Dermacentor marginatus*. (D) *Hyalomma marginatum*. The modelled habitat suitability of 11 species of vertebrates used as proof-of-concept is included in the Supplementary Material.

The algorithm best predicting the habitat suitability for each tick species was used to capture the set of variables chosen as the best predictive set. It is interesting to note that only the set of variables related to weather influenced the final models while the set of landscape-related variables was only secondary among the set of best descriptors. Each species had different landscape-related variables as better determinants of suitable habitat at the chosen scale, as shown in Table 4. Only the top 15 explanatory variables were included in the summary table. Supplementary Material 1 includes the complete set of variables and their relative contribution (measured by several indexes) to the best model obtained by the top-performing MLA. Most repeated features were related to the category of vegetation. In some cases, distance to impervious surface, buffer to grass areas, or type and category of forests were included in the models interpreted as an effect derived from the preferences of hosts. Importantly, no host-dependent variables were necessary to obtain the best models for the four target tick species. Interestingly, the selected tick species can be modelled without the inclusion of any vertebrate-derived variable. No MLA selected features regarding the habitat suitability for vertebrate species. Figure 1 shows the maps of predicted suitability (in terms of probability) for the four species of ticks modelled.

**Table 4.** The set of the explanatory variables that produced the better model for each species of tick.

	*I. ricinus*	*D. marginatus*	*D. reticulatus*	*H. marginatum*
VPD1	0.474	0.632	0.217	0.677
VPD_annual	0.468	0.686	0.204	0.658
TMax_annual	0.370	0.683	0.258	0.658
TCD_variance	0.341		0.106	0.070
TMax1	0.339	0.600	0.283	
TMin1	0.335		0.233	0.349
TMin_annual	0.306	0.415	0.223	0.448
VPD2	0.290	0.382	0.133	0.485
VPD3	0.288	0.174	0.096	0.343
Buffer_Imperv	0.250		0.194	0.064
EEA_maj	0.246	0.391	0.184	0.223
TMin2	0.244	0.189	0.167	0.060
EEA_median	0.238	0.356	0.158	0.227
TMax3	0.224	0.280	0.221	0.105
TMax2	0.198	0.150		
TMin3		0.207	0.145	0.100
TMax_annual		0.258		
Impervious surface		0.117		
EEA_min		0.114		
TCD_median				0.055

The abbreviations used in the table correspond with the definitions of each variable in Table 1. The color bars indicate the variables that have the greatest contribution in the modeling of each species of ticks.

Interestingly, most of the tick species., except *D. reticulatus*, have a similar set of important features, indicated in color in Table 4, as evaluated by the ‘information gain’ of the model after the selection of such feature(s). Therefore, most of the environmental niches of these ticks were dominated by a few prominent variables. The addition of other variables contributes to improving the predictive results, which is better seen for all species, except *D. reticulatus*, for which the contribution of each variable is poorer than for other species, probably indicating that the resolution of the dataset may not be suitable for this species.

### Vertebrates are better modelled by landscape-derived features

**Figure 2.**
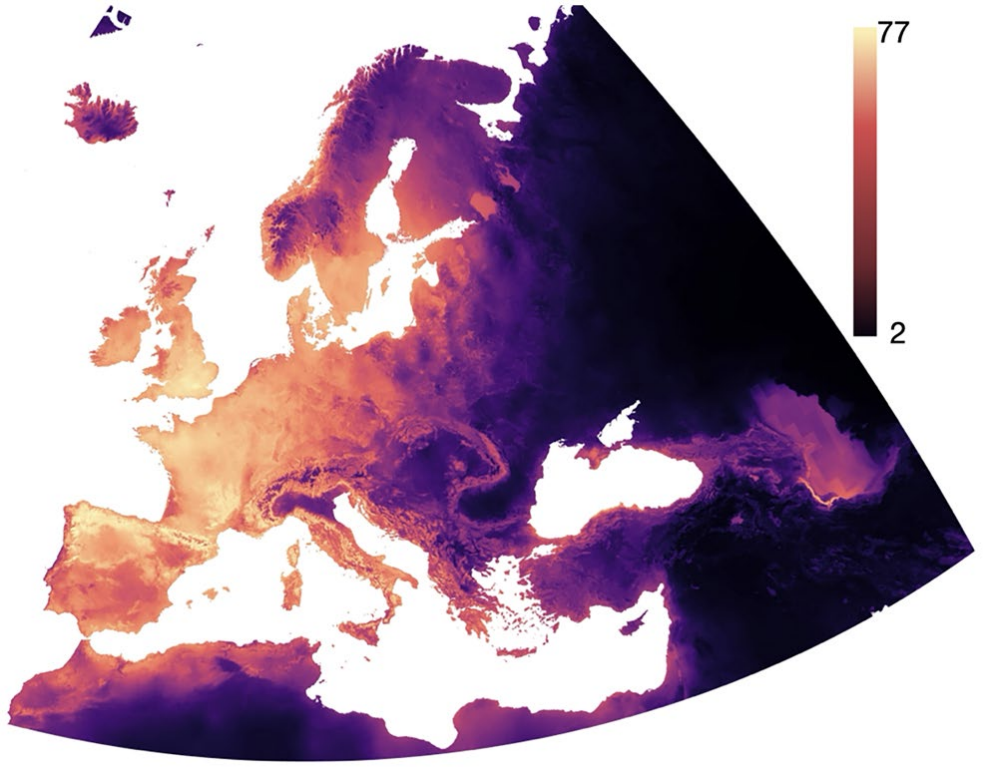
The species outcome (species diversity, beta diversity) of the vertebrates in the target area. The species outcome (species diversity, beta diversity) of the vertebrates in the target area. Wide areas of low species outcome over Russia and neighboring countries are not because these areas have low diversity of vertebrates. Models were produced for vertebrates with a European distribution range, therefore not colonizing territories at the east of the main target area of study (as shown in the figure).

As for the results obtained for ticks, three different MLA (Gradient Boosting, Random Forest, and Neural Networks) outperformed AdaBoost and Naive Bayes in the modelling of the environmental niche of vertebrate species that were tested as a proof-of-concept. Both AdaBoost and Naive Bayes performed ∼15% worse, using the indicators of performance as mentioned. All the values of AUC for each algorithm and the vertebrate species selected for the proof-of-concept are included in Table 5. Supplementary Material 2 includes the complete set of indexes of performance of the five algorithms, including CA, F1, Precision, Recall, and MCC, for these species, as well as the contribution of each variable to the best model. In general, the 162 vertebrate species were modelled with a similar performance (with a specificity near 85% and sensitivity higher than 93%) independent of the taxonomic status of the animal (mammal, bird, reptile), the number of available records (in every case more than 500 georeferenced records for the target territory), and its known range (widely distributed or restricted to a region). Figure 2 includes the species outcome of vertebrates in the target area; this can be quickly translated into beta-diversity of vertebrates in the region. Supplementary Material 3 includes maps of the expected environmental suitability for the 11 vertebrate species modelled as proof-of-concept. Other than the explanatory variables, the Geopackage distributed as Supplementary Material contains data of the predicted distribution of all species of mammals selected in the target territory.

**Table 5.** The values of AUC for each model of 11 vertebrate species modelled as a proof-of-concept.

Algorithm	Aa	As	Cc	Ce	Ee	Ll	Le	Sa	Pm	Ti	Tm	Tp
Gradient boosting	0.978	0.985	0.969	0.977	0.975	0.979	0.959	0.980	0.953	0.975	0.954	0.963
Random forest	0.978	0.984	0.970	0.977	0.977	0.979	0.963	0.980	0.955	0.976	0.960	0.967
AdaBoost	0.873	0.895	0.848	0.872	0.866	0.869	0.834	0.880	0.825	0.861	0.825	0.838
Naive Bayes	0.846	0.885	0.809	0.863	0.849	0.852	0.803	0.840	0.802	0.876	0.795	0.820
Neural network	0.976	0.986	0.961	0.976	0.972	0.981	0.953	0.975	0.948	0.972	0.945	0.957

Included are *Alces alces* (Aa), *Apodemus sylvaticus* (as), *Capreolus capreolus* (Cc), *Erinaceus europaeus* (Ee), *Lacerta lepida* (Ll), *lepus europaeus* (Le), *Sorex araneus* (Sa), *Parus major* (Pm), *Turdus iliacus* (Ti), *Turdus merula* (Tm), and *Turdus philomelos* (Tp).

## Discussion

The objective of this study was twice, namely (i) the building of a dataset including the largest number of variables with ecological meaning that could be of interest for studies regarding the epidemiology of ticks and tick-borne pathogens, and (ii) to elaborate on the ability of MLA to predict the range of the most important species of ticks (and their hosts) affecting human health in a large territory like Europe. The distribution of the vertebrates that act as hosts for the ticks or reservoirs for the pathogens they transmit is a concept of interest in a concept for increasing the impact of machine learning to face infectious disease challenges [26], global change, and the spread of TBPs affecting human health worldwide [1]. Automatic algorithms can systematically ingest information and produce ‘risk’ maps, with a focus on preventing the transmission of TBPs to humans. To achieve this objective, we built an integral dataset of climate, vegetation, and landscape features for the European territory, obtained from authorized sources of official institutions, and conforming a grid over the target territory. Such dataset is intended as the backbone of future research on the topic demonstrating its capabilities to capture the environmental niche of the targeted organisms. The final purpose is to improve human health since ticks are vectors of many human-threatening microorganisms. Predictive mapping and evaluation of climate change scenarios (affecting the phenology and distribution of vertebrates) seem to be the best ways to achieve such active prevention.

The dataset includes (a) known physical (landscape) parameters affecting the distribution of ticks and their hosts, (b) the predicted distribution of 162 species of vertebrates that have been recorded as hosts for the ticks in the target territory, and (c) climate data, including maximum and minimum temperatures and a measure of humidity in the air, which is missing in many studies regarding the expected habitat suitability for ticks [22]. Studies have addressed the importance of these variables in the modeling of tick environmental suitability [27]. We selected a grid format delivered in a GeoPackage file to incorporate the explanatory variables, in a common and open format, compatible with the current standards allowing sharing information associated with spatial structures; these variables are commonly available in a variety of other formats that could make it difficult to model over such a heterogeneous framework. It has been demonstrated that, at least for biological studies, a hexagonal design like the one produced in this study summarizes better the habitat structure in the territory covered by each cell [28]. This is the largest effort to produce a massive dataset for mapping purposes; as such, other algorithms or modelling approaches have not been ever tested on the set of data released in this study.

In recent decades, efforts have been made to apply a variety of methods to the prediction of the environmental niche of ticks and TBPs [27, 29–31]. These strategies, however, has been criticized because of the poor choice of explanatory variables, that are expected to not reflect correctly the climate niche of the ticks, and/or the systematic use of a few methods of modelling [32, 33] without testing new possibilities. One of the main problems is the use of interpolated climate data originally conceived to describe Earth’s climate. At least in the case of ticks, the choice of the variables for modelling exercises has been generally carried out by researchers after the subjective removal of some variables [34] based commonly on autocorrelation features. However, the elimination of these variables could eliminate the most prominent features explaining the actual distribution of the target organism. It has been demonstrated [32] that harmonic regression of temperature values produces a few variables that retain the maximum explanatory power. The first three coefficients of a harmonic regression can describe the seasonal variation of the variable, without the need for pre-tailored datasets [32] and accounting for an ecological perspective regarding the organism to be modelled. Other sources of unreliability commonly consider that the distribution of TBPs can be predicted using only the distribution of tick vectors (as mentioned in e.g. [33]). Ticks and reservoirs shape finely tuned biotic combinations that are behind major changes in pathogen prevalence across European biotopes [33, 35, 36]. Nevertheless, it is still far from completely understanding the phenomena that erect vertebrate communities [37, 38], and this is why we assumed a species-by-species model instead of a joint model of the communities.

Our results showed that models of tick distribution were not improved by the inclusion of the distribution of hosts. This is important in the modern context of the epidemiology of TBPs because may be indicative of the probable lack of importance of vertebrate species [39, 40]. Results are suggestive that key hosts may be necessary for the circulation of selected TBPs [39]. A *continuum* of species-abundance patterns of the community of vertebrates, that changes across the space, could thus be responsible for the support of most TBPs. These communities of vertebrates would distribute along patterns of climate and landscape variables, replacing one to each other in the space promoting a gradient of TBPs circulation. Under this hypothesis, the abrupt decline or absence of several vertebrates simultaneously would be responsible for the absence of the pathogens. The challenge is the modeling of the tick distribution against the groups of vertebrate’s communities. We consider that although an improved modeling framework could be an efficient method to address the impact of key hosts on tick’s distribution patterns, only field surveys can produce the necessary data supporting the importance (even at local scales) of different species of hosts in the mixture of a community assemblage.

The largest approach to a European-wide modeling of several tick species has been addressed previously using null-models and looking for a clear climate signal shaping the predicted tick distribution [41]. This study concluded that only one tick species (*Hyalomma lusitanicum*, not included in the current study) did not show a strong climate signal and elaborated on the probable role of vertebrates in shaping the niche of the tick. While we consider that the modeling background developed in that previous study is solid and coherent, we wonder if the few records of the tick available in the compilation used by the authors produced the confusing results as reported [41]. We interpret these findings as the dimensions of a hypervolume formed by the number of explanatory variables (features). Each tick species colonizes different portions of the dimensions of such hypervolume shaped by climate and landscape variables. The axes of the hypervolume of TBPs could include both the ‘basic’ set of variables defining tick distribution, plus the ones derived from vertebrates, a concept that has been approached on several occasions in ecology and epidemiology [42]. Attention must be paid to the fact that such a concept would be lost if a reduction in principal components is used to simplify the number of explanatory variables (as reported in Refs. [43, 44]).

It is difficult to model the distribution of vertebrates at such a large scale because of several possible gaps, such as (a) the different collection pressures carried out at different sites in Europe, producing heterogeneous collections of records, (b) the status of some species as protected or of difficult trapping, rendering few georeferenced records to train models, or (c) the scientific interest of a species in the circulation of pathogens of any kind that increases its surveillance, thus resulting in an extra number of records relative to other species. All these effects produce a bias in the known distribution of species. As expected, the vertebrates included in this study were better modelled with combinations of landscape-derived variables, such as the type of forest, the buffer effect of some vegetation types, or the distance to human shelters or impervious areas. Although climate may play a role shaping the distribution of some species, the rule observed is that vertebrates are mainly affected by landscape features. This has an effect that pervades the modelling of ticks because landscape-derived variables have also been selected in tick models, although ranked in less prominent positions. The expected distribution of ticks was adequately captured [45] but we noticed a critical gap in modelling the distribution of *D. reticulatus*. It is well known that *D. reticulatus* permanently colonizes regions east of the area calculated by our models [46]. This underprediction has not been captured by the measures of sensitivity and specificity of the model. This points to an issue borne in the calibration of the model with insufficient data or without reliable records [44, 45]. We consider the most probable source of error to be the coarser resolution used in the current study. It has been reported (e.g. [46–48]) that this tick may occur in small patches because local conditions that could not be captured by the rough resolution of the grid dataset. This is something that should be considered in future developments, aiming to conciliate the different modelling approaches with particularly complex species.

Our results cannot support the recently revisited modeling of *H. marginatum* in its range [49]. The expected distribution of such prominent tick species has been reported several times [50–53] and these outcomes overlap well with the expected resolution of the tick as shown in our study, even if developed at low resolution and using radically different algorithms. We consider that the unexpected results reported for *H. marginatum* in Ref. [49] in which a Mediterranean tick was predicted to have suitable habitat in, e.g. northern Scotland and most of central Europe resulted from the poor choice of records (including those reported as ‘invasive’ in central and northern Europe, and unverified records disconnected from the main population area) and the incorrect selection of explanatory variables. Given the importance of *H. marginatum* in human health, we consider urgent the verification of these results [49] that are not congruent with any other approach to the known distribution and modeling of the species. The lack of Supplementary Material in the original publication [49] makes unfeasible any corroboration. In any case, it results complex the comparison of the MLA modelling approach with other methods commonly used for tick distribution modelling [13, 19, 29, 32]. A comparison of methods has not been carried out, because other methods have been commonly driven by explanatory layers as raster data. Our approach has been the opposite: summarizing explanatory variables in coherent blocks of information that are statistically tractable by solid statistics. Future studies should address comparative modelling results, and field studies about vertebrate communities, climate, and ticks, would confirm the findings predicted in the current approach. The deep study of the many ‘combinations’ of hosts and ticks, together with an accurate dataset of pathogen’s prevalence in ticks and hosts for the whole territory (with different species of vectors and reservoirs) is ongoing

The spread of ticks affecting human health and their impact on the world health system has enhanced efforts to predict and anticipate their impact on human health. Reliable and precise methods are necessary to handle the variable, non-linear interactions among variables since these variables define (a) the ecological niche of organism(s) and (b) the areas of risk for humans (once translated into a map). We consider that MLA can be used in a chain of decisions aimed at improving these aspects of human health. The results of these models can be applied to a better design of preventive and control interventions for TBP, such as personalized medicine-based vaccines and therapeutics.

## Ethical approval

This study did not involve human, animal, or tissue experimentation.

## Consent for publication

Not applicable.

## Supplementary Material

Supplemental Material

## Data Availability

All the data used in the preparation of the study are available in the manuscript or on the web mentioned under ‘Supplementary Material’.
