# Supplementary material for: Machine learning algorithms for the evaluation of risk by tick-borne pathogens in Europe
Source: Ann Med. 2024 Sep 30;56(1):2405074. doi: 10.1080/07853890.2024.2405074 (PMC11443563; doi:10.1080/07853890.2024.2405074)
Supplement: Supplemental Material [file IANN_A_2405074_SM6589.docx]

**Supplementary Material**

The Supplementary material includes the performance of the models developed for ticks and vertebrates (Supplementary Data 1 and 2, respectively). Supplementary Material 3 includes figures of habitat suitability for the 11 vertebrate species selected as proof-of-concept (the figures are labelled with the names of the species). The complete dataset is in GeoPackage format, as Supplementary material 4. The Supplementary material 4 contains a metadata file with a description of all the columns of the sheet associated with the GeoPackage, which opens with most modern open-access GIS tools, including (but not exclusive to) qGIS. This is also readable by most commercial GIS packages. Scripts of the modelling framework are also provided as Supplementary material 5 (ticks) and 6 (vertebrates), as used in this study. The scripts contain tests with different species, as examples of the programming framework; they are not intended for completeness, but modelling of every species could be achieves with simple copy/paste of the necessary icons (algorithms) in the script. They run under the free software Orange (available at https://orangedatamining.com). Supplementary material can be found at 10.6084/m9.figshare.25197437.
